# Atomic Layer Deposition of Intermetallic Fe_4_Zn_9_ Thin Films from Diethyl Zinc

**DOI:** 10.1021/acs.chemmater.2c00907

**Published:** 2022-05-23

**Authors:** Ramin Ghiyasi, Anish Philip, Ji Liu, Jaakko Julin, Timo Sajavaara, Michael Nolan, Maarit Karppinen

**Affiliations:** † Department of Chemistry and Materials Science, Aalto University, FI-00076 Espoo, Finland; ‡ Tyndall National Institute, UCC, Cork T12 R5CP, Ireland; § Department of Physics, University of Jyväskylä, FI-40014 Jyväskylä, Finland

## Abstract

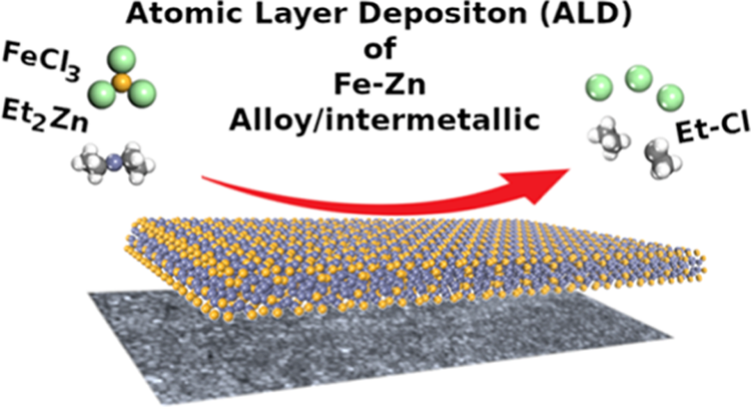

We present a new type of atomic layer deposition (ALD) process for intermetallic thin films, where diethyl zinc (DEZ) serves as a coreactant. In our proof-of-concept study, FeCl_3_ is used as the second precursor. The FeCl_3_ + DEZ process yields in situ crystalline Fe_4_Zn_9_ thin films, where the elemental purity and Fe/Zn ratio are confirmed by time-of-flight elastic recoil detection analysis (TOF-ERDA), Rutherford backscattering spectrometry (RBS), atomic absorption spectroscopy (AAS), and energy-dispersive X-ray spectroscopy (EDX) analyses. The film thickness is precisely controlled by the number of precursor supply cycles, as expected for an ALD process. The reaction mechanism is addressed by computational density functional theory (DFT) modeling. We moreover carry out preliminary tests with CuCl_2_ and Ni(thd)_2_ in combination with DEZ to confirm that these processes yield Cu–Zn and Ni–Zn thin films with DEZ as well. Thus, we envision an opening of a new ALD approach based on DEZ for intermetallic/metal alloy thin films.

## 1 Introduction

Atomic layer deposition (ALD) is the fastest growing thin-film technology in microelectronics and beyond, owing to the superior film characteristics it provides.^1−6^ In a prototype ALD process, two different gaseous/vaporized precursors are sequentially pulsed and purged out of the reaction chamber with prespecified time intervals.^7,8^ The precursor pulse and purge times are selected so that the precursors have enough time to chemisorb on the surface and react with the available surface groups for full surface coverage. Another important feature is the self-limitation of the surface reactions such that the chemisorption of one precursor is limited to a monolayer; the reaction only continues after the excess precursor molecules from the gas phase are purged out and the second precursor is delivered to the reaction chamber. As the two precursors are not present at the same time in the chamber, no unwanted gas-phase reactions occur. This unique growth process leads to highly uniform and conformal thin films with atomic-level control of both thickness and composition.

Most commonly the ALD technique is applied to the fabrication of binary metal oxide (e.g., Al_2_O_3_, HfO_2_, TiO_2_, ZnO),^9,10^ sulfide (e.g., ZnS),^1,11^ or nitride (e.g., Si*_x_*N*_y_*)^12^ thin films; in these depositions, the second precursor is the source of oxygen (e.g., H_2_O, O_3_), sulfur (e.g., H_2_S, S), or nitrogen (e.g., NH_3_). Ternary and even quaternary processes are possible as well, but these are more challenging to optimize.^13−15^ More recently, the interest in ALD of pure metals has been rapidly increasing.^16,17^ Here, the major challenges are related to finding the optimal conditions for the reduction of the metal cations and tackling the agglomeration/island growth issues, leading to rough and even discontinuous films. In the case of noble metals, specific reductants are not always needed; a renowned example in combustion reactions of platinum-group metal precursors is oxygen.^18^ For the non-noble metals, molecular H_2_ is the most common reducing agent.^19^ However, its limited reactivity is a major challenge. Hydrogen plasma is another possibility,^20^ but a common difficulty is the film conformality. More recently, different organic reactants have been investigated as well for the growth of metal films.^21,22^

Considering the challenges with the ALD fabrication of single-metal thin films, it is not surprising that the application of ALD in depositing multimetal (metal alloys or intermetallic compounds) is limited to a few successful examples only.^23−26^ The highlights in this area include the pioneering work of Christensen and Elam^23^ in depositing Ir–Pt alloys from Ir(III) acetylacetonate/O_2_ and (trimethyl)methylcyclopentadienyl Pt(IV)/O_2_ cycles, and the more recent successes by Väyrynen et al.^24−26^ in depositing Co_3_Sn_2_, Ni_3_Sn_2_, and Ni_2_Ge films using carbene metal hydrides with a metal–hydrogen bond as a reductant. Hydrides have also been used to deposit single-metal Al films.^27^

Herein, we introduce a novel ALD approach not relying on hydrogen or hydride species for multimetal thin films. The process is based on one of the most common ALD precursors, that is, diethyl zinc (DEZ) extensively used for the growth of ZnO films.^10^ It should be noted that there are a couple of reports on the use of DEZ as a reductant for pure metallic films;^28−31^ interestingly, depending on the deposition temperature, some unintended inclusions of Zn deposits in the Cu films were observed in these films occasionally. Now, we demonstrate that when combining DEZ with FeCl_3_—another well-behaving ALD precursor—^32,33^^32,33^ we can deposit in situ crystalline iron–zinc intermetallic films in a controlled manner. Here, we may see some similarities to the works by Xiang et al.^34−36^ who deposited Ti–Al films from trimethylaluminum (TMA) and TiCl_4_. However, in these films, the carbon contamination was so high that the films were closer to the Ti–Al–C carbide composition. We also like to mention that in another somewhat similar process, Pore et al.^37^ deposited Sb and Ge telluride films from chloride and trialkylsilyl precursors.

There are five intermetallic compounds reported in the Zn–Fe system, in the order of increasing Fe content: ζ (FeZn_13_), δ1p (Fe_13_Zn_126_), δ1k (FeZn_7_), Γ1 (Fe_11_Zn_43_), and Γ (Fe_4_Zn_9_).^38,39^ Among these, the γ-Fe_4_Zn_9_ phase with the highest Fe content and a bcc-type crystal structure is probably the best established one and also the product of our ALD process. In the literature, the γ-Fe_4_Zn_9_ phase has been reported to form with the lower Fe/Zn ratios of FeZn_3_ and Fe_3_Zn_10_ as well.^38,39^ This seems to be the case with the present study too. In automotive and other industries, Fe–Zn layers are conventionally produced through electroplating on galvanized steel or other metal surfaces, e.g., for anticorrosive or mechanical coatings.^40−42^ More recently, Zn–Fe coatings have been investigated, for example, as biodegradable medical coatings on implants,^43,44^ where the ALD technique could offer unique advantages.

## 2 Experimental Section

Fe–Zn thin films were deposited in a flow-type thermal ALD reactor (ASM Microchemistry F120) on 2 × 2 cm^2^ glass and silicon substrates. For the depositions, the FeCl_3_ precursor powder (Merck, 95%) was placed inside the reactor and heated at 158 °C for sublimation, while the DEZ precursor (Zn(CH_2_CH_3_)_2_; Sigma-Aldrich, ≥52 wt % Zn basis) bottle was kept outside the reactor at room temperature. Nitrogen (99.999%; N_2_ 5.0) was used as the carrier and purge gas. It should be emphasized that no other reactants were employed, and the reactor was kept under a constant vacuum (2.6 mbar). The depositions totaled a fixed number of cycles (100, unless otherwise stated), and each cycle consisted of the following four gas pulses: DEZ precursor, N_2_ purge, FeCl_3_ precursor, and N_2_ purge. The purge time was always twice the precursor pulse time to make sure that all of the possible leftover materials from the previous pulse were eliminated from the gas phase; to be sure that this purge length was long enough, we investigated also shorter (1.5 times) and longer (4 times) purges to confirm that the film growth rates remained the same.

Profilometry (Veeco Dektak 6M stylus profilometer) and X-ray reflectivity (XRR; Panalytical XPert diffractometer, Cu Kα source) methods were utilized to determine the film thicknesses. The surface morphology was studied by scanning electron microscopy (SEM; Hitachi S-4700). The crystallinity and phase composition of the films were investigated by grazing incidence X-ray diffraction (GIXRD; incidence angle of 0.5°; the same diffractometer as for XRR).

The chemical composition of the films was addressed through several different approaches—using both easily accessed in-house techniques and more advanced characterization tools—to get a comprehensive view, as the different techniques were assumed to complement each other regarding the information they provide. Atomic absorption spectroscopy (AAS; Varian AA240) was used as the first tool to routinely check the samples for the Fe/Zn ratio. For the AAS analysis, the films were deposited on a glass substrate from which the film material could be quantitatively dissolved in nitric acid, followed by dilution with water; each analysis was repeated three times to obtain the Fe/Zn ratio with an appreciable certainty. Some of the samples were also investigated during the initial process development to rule out the Cl contamination (and also for the semiquantitative Fe/Zn ratio) by energy-dispersive X-ray spectroscopy (EDX; Tescan Mira 3). In the EDX measurements, the elemental mapping was carried out on the entire substrate to also address the homogeneity of the films. Later, the Fe/Zn ratios were measured using Rutherford backscattering spectrometry (RBS) and time-of-flight elastic recoil detection analysis (TOF-ERDA) for a selection of films grown on silicon substrates. Most importantly, from the ERDA measurements, the light-element (C, O, Cl) contamination level could be accurately addressed. The RBS measurements were carried out by the application of a ^4^He^1+/2+^ beam with 2 and 3 MeV energies and TOF-ERDA measurements using an 11.9 MeV ^63^Cu^7+^ ion beam from a 3 × 3 mm^2^ surface area.

**Figure 1:**
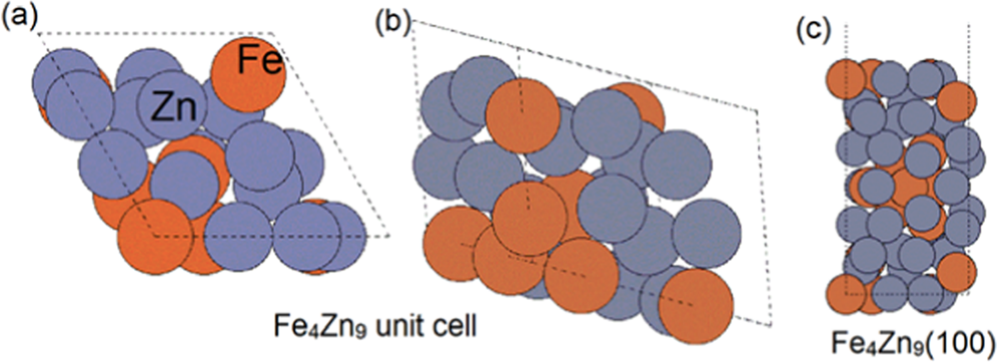
Configurations of (a) top view and (b) side view of the Fe_4_Zn_9_ unit cell and (c) Fe_4_Zn_9_(100); zinc and iron atoms are represented by dark gray and orange colors.

To address the surface reactions, computational calculations were performed on the basis of periodic spin-polarized density functional theory (DFT) within a plane-wave basis set and projector augmented wave (PAW) formalism, as implemented in the Vienna Ab initio Simulation Package (VASP 5.3) code.^45^ The generalized gradient approximation (GGA) with the parameterization of Perdew–Burke–Ernzerhof (PBE) was used for the exchange–correlation functional.^46,47^ The plane-wave energy cutoff was set to be 400 eV. The valence electrons were 12 for Zn, 8 for Fe, 7 for Cl, 4 for C, and 1 for H. The convergence of energy and forces were set to be 1 × 10^–4^ eV and 0.02 eV/Å, respectively. A *k*-point mesh^45^ of 3 × 3 × 1 was used throughout the calculations. The unit cell of Fe_4_Zn_9_, shown in Figure 1, was obtained from Material Project.^48^ The lattice constant was *a* = *b* = *c* = 7.77 Å. The slab model of Fe_4_Zn_9_(100) had 20 Fe atoms and 40 Zn atoms, where all of the atoms were allowed to relax. A 15 Å vacuum region was applied. The molecular geometries of precursor FeCl_3_ and DEZ (Zn(Et)_2_) and byproducts were relaxed in the same supercell as the slab model of Fe_4_Zn_9_, with an energy cutoff of 400 eV and γ point sampling. The van der Waals correction was applied with the PBE-D3 method to ensure an accurate description of the metal precursor adsorption energy. The adsorption energy was calculated from
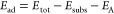
where *E*_tot_, *E*_subs_, and *E*_A_ are the energy of the slab with precursors, the slab model for Fe_4_Zn_9_, and isolated precursors or the byproduct, respectively.

## 3 Results

**Figure 2:**
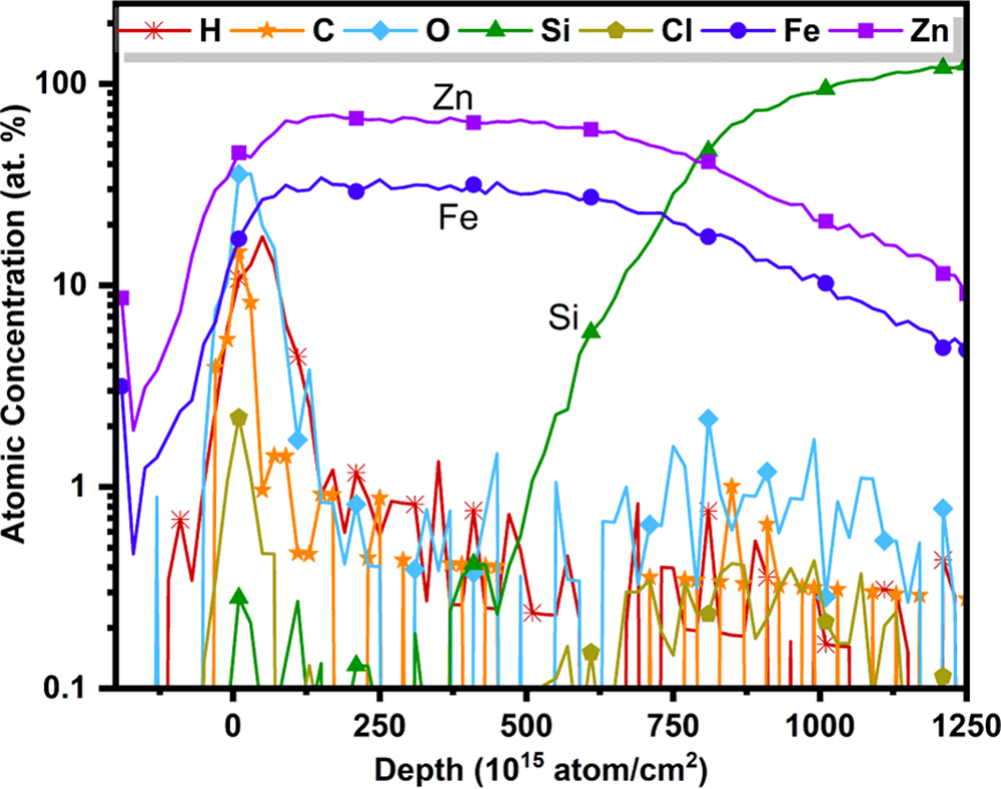
Elemental depth profile obtained with TOF-ERDA, for example, the thin-film sample deposited at 260 °C.

All our depositions from DEZ and FeCl_3_ yielded visually homogeneous crystalline thin films for which no other elements than zinc and iron were detected from the EDX analysis. The homogeneity was additionally confirmed by the EDX mapping over the entire surface area. For representative samples, the light-element (C, O, Cl) impurity levels were analyzed in detail using TOF-ERDA, see Figure 2 for a depth profile. On the very surface of the films, TOF-ERDA detects of C, O, and Cl (presumably due to the incomplete loss of precursor ligands) were observed, but deeper in the bulk, the films have impurity concentrations well below 1 atom % for H, C, and O. From both AAS and RBS analysis, all of the films contained iron and zinc, the Fe/Zn ratio slightly varying depending on the deposition conditions. The GIXRD patterns indicated that γ “Fe_4_Zn_9_” was the main phase in all samples, with a minority Fe metal phase in the Fe-rich region. No indication of Zn metal was seen for any of the samples; this is an important note, as DEZ has been seen to decompose into metallic Zn in some ALD processes at high temperatures and with long DEZ pulses.^49^ Also, we confirmed that no film growth occurred when DEZ was pulsed alone into the reactor.

**Figure 3:**
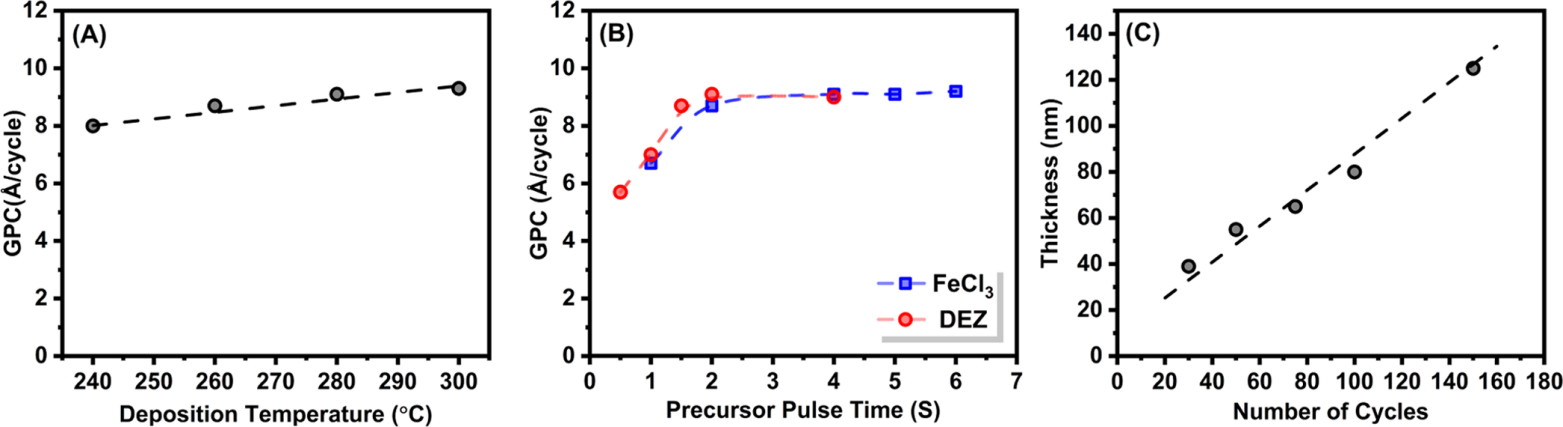
Characteristics of the DEZ + FeCl_3_ process: (A) GPC value versus deposition temperature, (B) GPC value versus precursor pulse lengths for both precursors, and (C) film thickness versus the number of deposition cycles. In (A) and (B), the number of cycles was 100. In (B) and (C), the deposition temperature was 260 °C; the GPC values are from profilometry data.

We investigated our DEZ + FeCl_3_ process systematically against various deposition parameters; the results are summarized in Figure 3. The influence of the deposition temperature on the growth-per-cycle (GPC) value is shown in Figure 3A for the range of 240–300 °C; outside of this temperature range, the films were found to be nonuniform. In these experiments, the precursor pulse lengths were kept unchanged at 2 s DEZ and 2 s FeCl_3_. The tiny increase in GPC with temperature seen in Figure 3A could be explained by the fact that we also observed minor changes in the Fe/Zn ratio in AAS measurements, the Zn content slightly increasing with increasing deposition temperature.

One of the cornerstones of an ALD process is the saturation of the surface reactions within each precursor pulse. This is typically demonstrated by showing that the GPC value first increases and then saturates upon increasing the precursor pulse lengths. In Figure 3B, we demonstrate this for our DEZ + FeCl_3_ process by plotting the GPC value against the pulse lengths of the two precursors, one varied at the time, while keeping the other fixed (at 4 s for FeCl_3_ and at 2 s for DEZ). These experiments were carried out while keeping other process parameters fixed, i.e., the deposition temperature at 260 °C and the number of cycles at 100. It should be noted that we carried out additional experiments where we tested other pulse length combinations as well to verify that the saturation behavior for one of the precursor pulse lengths was not sensitively depending on the choice of the pulse length for the other. From Figure 3B, we can see that the initially chosen pulse lengths of 4 s for FeCl_3_ and 2 s for DEZ are long enough to reach saturation. The growth rate saturates to the relatively high GPC value of ca. 9.0 Å/cycle, which is—interestingly—close to the lattice parameter (8.982 Å)^42^ of the γ-Fe_4_Zn_9_ phase. This is likely to result from the highly exothermic reactions involved in the deposition of FeZn from FeCl_3_ and DEZ, as described in Section 4.

Another important criterion for an ALD process is the growth linearity in terms of the number of precursor pulsing cycles applied. Figure 3C illustrates this for the present FeCl_3_ + DEZ process. For these experiments, the precursor pulse lengths were fixed to 4 s for FeCl_3_ and 2 s for DEZ, and the deposition temperature was 260 °C.

**Figure 4:**
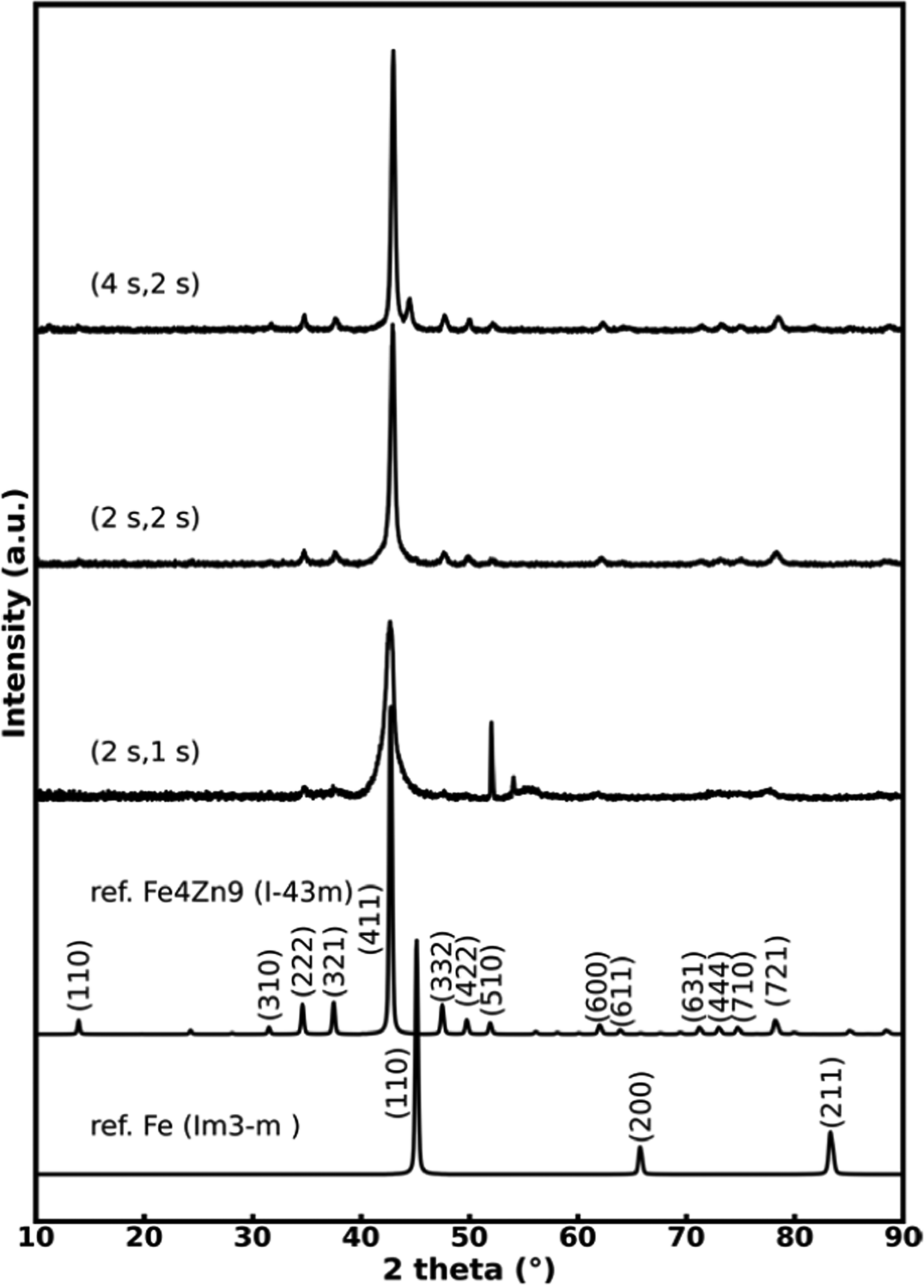
GIXRD patterns for the Fe–Zn films grown at 260 °C with different precursor pulse lengths, together with indexed reference XRD patterns for γ-Zn_9_Fe_4_ and α-Fe; peaks in the range seen for the (2 s, 1 s) sample in the 50–60° area are due to the Si substrate.

We then characterized the resultant Zn–Fe films for their elemental and crystalline phase compositions. In Figure 4, GIXRD patterns are displayed for three representative 78–93 nm thick samples, all deposited at 260 °C but with somewhat different precursor pulse lengths, together with simulated reference patterns for γ-Fe_4_Zn_9_ and α-Fe.^50,51^Table 1 lists the atomic Fe/Zn ratios for the same samples as obtained from the RBS, AAS, and EDX analyses. Among the three techniques, RBS is the most accurate, while EDX is considered only semiquantitative, and moreover surface sensitive. The absolute values from the different techniques do not match perfectly but the trends are quite similar though, i.e., the Fe content increases with increasing precursor pulse lengths. Here, we should recall that among the three samples, only the one with the longest precursor pulses (4 s FeCl_3_, 2 s DEZ) was grown in an area clearly fulfilling the surface saturation conditions (Figure 3).

**Table 1:** Atomic Fe/Zn Ratio Estimated from RBS, AAS, and EDX Analyses for the Fe–Zn Films Grown at 260 °C with Different Precursor Pulse Lengths

sample (FeCl_3_, DEZ)	RBS	AAS	EDX
(4 s, 2 s)	0.51	0.39	0.6
(2 s, 2 s)	0.19	0.32	0.5
(2 s, 1 s)	0.12	0.15	0.2

From Figure 4, while the Fe_4_Zn_9_ phase dominates all of the three GIXRD patterns, the film deposited with the shortest precursor pulse lengths (2 s FeCl_3_, 1 s DEZ) shows the broadest diffraction peaks, i.e., the lowest degree of crystallinity. Also, the elemental Fe/Zn ratio for this sample is clearly below the “stoichiometric” value of 0.44 assumed for Fe_4_Zn_9_ (although based on the previous literature, the Fe_4_Zn_9_ phase may be stabilized over a relatively wide compositional range, even down to the Fe/Zn ratio of 0.30).^38,39^ On the other hand, for the sample deposited with the longest precursor pulse lengths (4 s FeCl_3_, 2 s DEZ), the values obtained for the Fe/Zn ratio with the different analysis techniques are quite close to the ideal 0.44 value (Table 1). However, for this sample, a trace of the α-Fe phase is distinguished from the GIXRD pattern. The highest Fe_4_Zn_9_ phase purity is seen for the sample deposited with the pulse lengths 2 s for FeCl_3_ and 2 s for DEZ. The density value determined for this highly crystalline and essentially single-phase Fe_4_Zn_9_ sample from the XRR fitting (7 g/cm^3^) is quite close to the ideal value of 7.44 g/cm^3^ calculated from the crystal structure data for γ-Fe_4_Zn_9_. The Fe/Zn value (from the most accurate RBS/TOF-ERDA analysis) for this sample appears a little low against the nominal Fe_4_Zn_9_ stoichiometry; unfortunately, the reason for this could not be clarified within this study.

**Figure 5:**
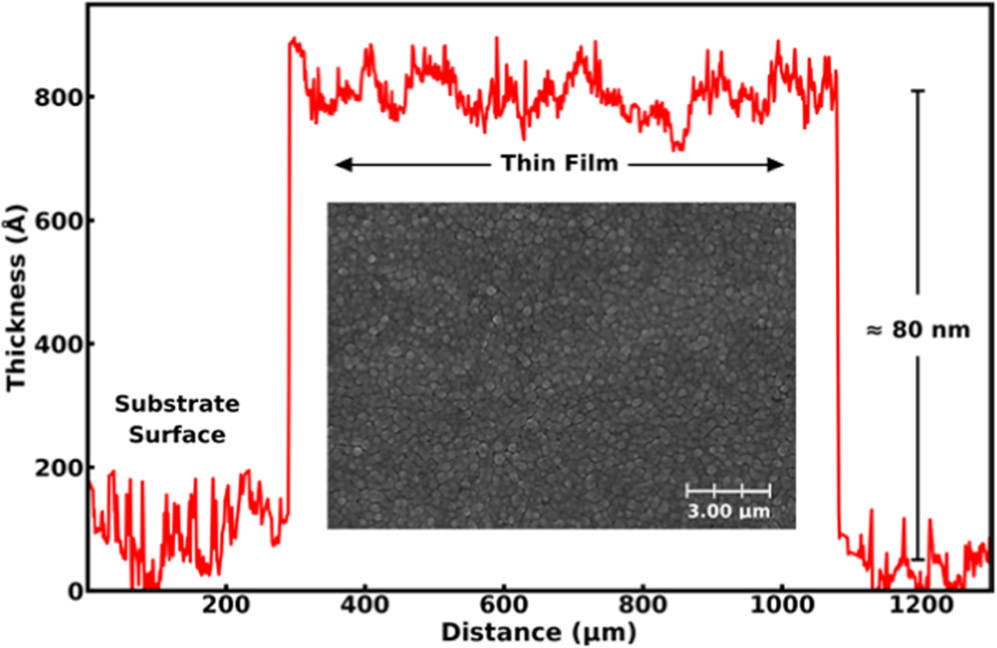
Surface profile and the SEM image for the Fe–Zn film deposited at 260 °C (2 s, 2 s).

We also addressed the surface morphology of the most phase-pure and highly crystalline sample by measuring its surface profile using profilometry (Figure 5). The film thickness is obtained at ca. 80 nm in good agreement with the XRR-based estimation, and the small fluctuations detected in the profilometry data on the film surface are very similar to those seen on the substrate surface, indicating that the film growth itself yields smooth films. The SEM image for the same sample is displayed in the inset, showing the homogeneous nature of the films. The grains are well dispersed with an average size of 84 ± 12 nm.

Finally, we like to mention that we carried out very preliminary experiments for other metal precursors in combination with DEZ and observed that similar processes with CuCl_2_ and Ni(thd)_2_ yielded multimetal (Cu–Zn and Ni–Zn) films as well. Hence, it tentatively seems that the presence of DEZ plays an important role in these processes.

## 4 Discussion on the Mechanism

**Figure 6:**
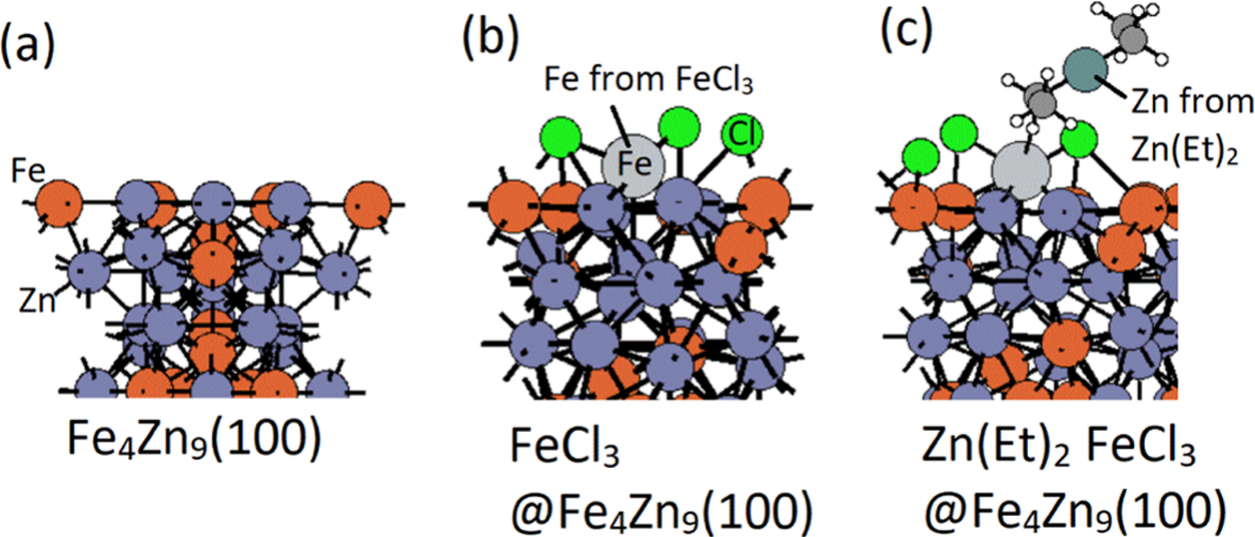
Structures of (a) slab model of Fe_4_Zn_9_(100), (b) FeCl_3_ adsorption on Fe_4_Zn_9_(100), and (c) Zn(Et)_2_ and FeCl_3_ coadsorption on Fe_4_Zn_9_(100). Substrate Zn and Fe atoms are represented by dark gray and orange colors, Zn and Fe from precursors by dark green and light gray colors, and C, H, and, Cl by black, white, and green colors.

**Scheme 1:**
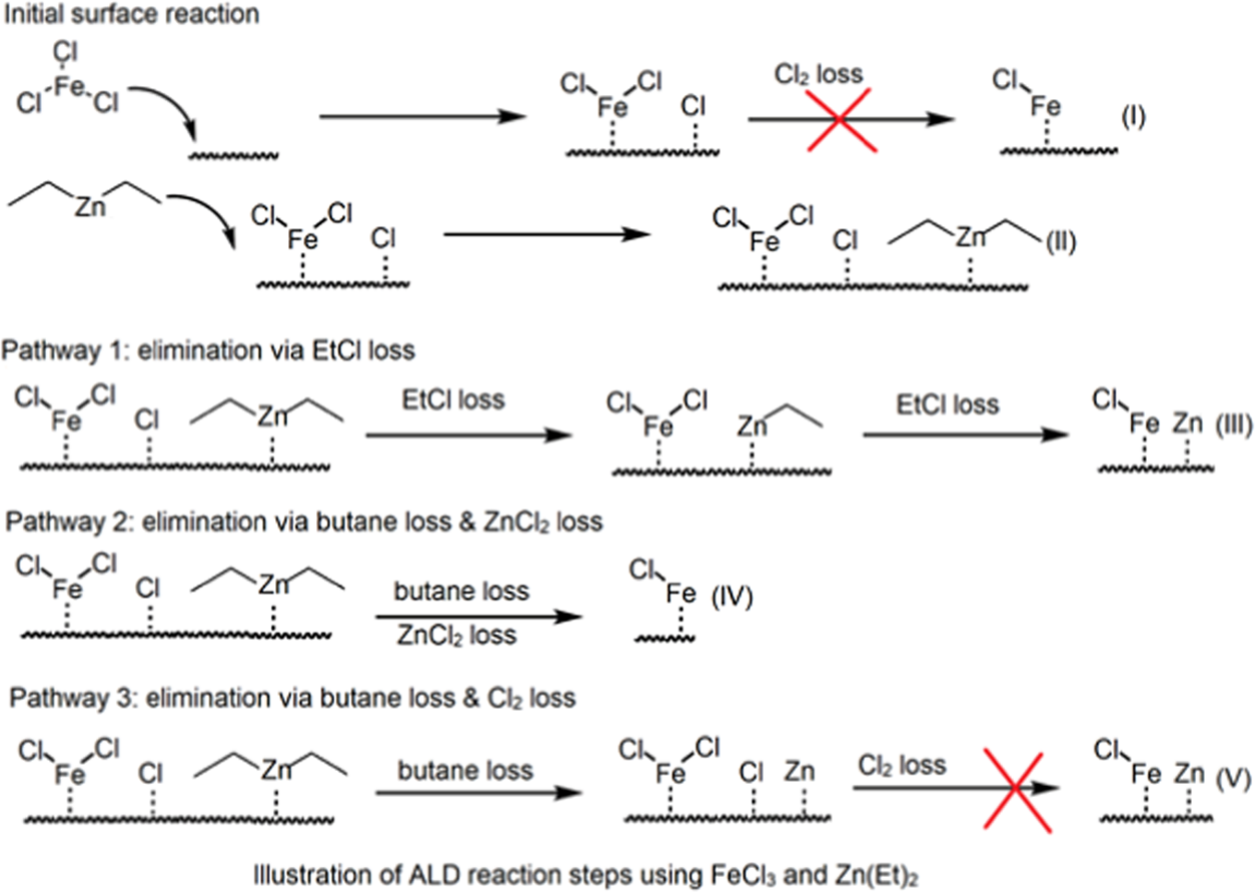
Illustration of the Proposed Reaction Steps between FeCl_3_ and Zn(Et)_2_ (I) Initial surface reaction of FeCl_3_ results in −FeCl_2_ and −Cl on the surface. (II) Initial surface reaction of ZnEt_2_ results in physisorption of Zn(Et)_2_. (III) Ligand elimination pathway via EtCl loss. (IV) Ligand elimination pathway via butane loss and ZnCl_2_ loss. (V) Ligand elimination pathway via butane loss and Cl_2_ loss.

We discuss the film growth mechanism based on the DFT calculation results. The proposed plausible ALD reaction steps are shown in Scheme 1; upon adsorption, FeCl_3_ has strong exothermic adsorption energy at −4.85 eV, resulting in bond breaking of Fe–Cl. The direct Cl_2_ loss has a high energy cost at a value of 4.69 eV. This direct Cl_2_ loss is not considered an elimination pathway. Zn(Et)_2_ is added to the surface, resulting in exothermic reaction energy at −5.87 eV. The structures of FeCl_3_ adsorption and Zn(Et)_2_ adsorption on the Fe_4_Zn_9_(100) surface are shown in Figure 6.

**Figure 7:**
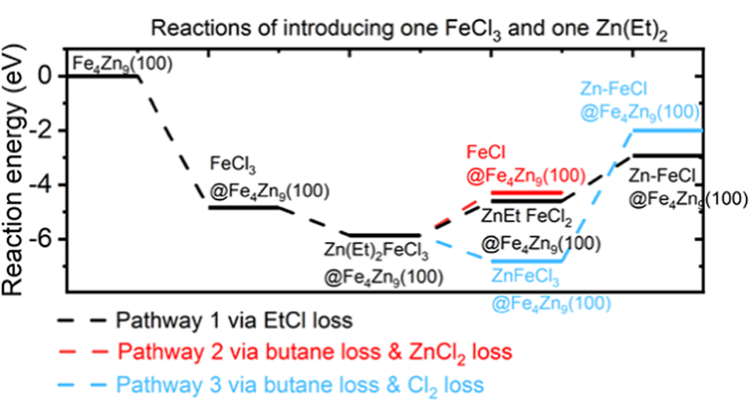
Plotted reaction energy pathways for eliminating the Cl group and the Et ligand via pathways 1, 2, and 3.

We then simulated three plausible pathways, as shown in Scheme 1, and plotted the corresponding reaction pathways in Figure 7. We first focused on introducing one FeCl_3_ and one Zn(Et)_2_ precursor. Pathway 1 via EtCl loss is the most exothermic reaction pathway, with computed overall reaction energy at −4.61 eV for the first EtCl loss and −2.94 eV for the second EtCl loss. Pathways 2 and 3 contain butane loss. For pathway 2, in addition to butane, a Cl group is eliminated via ZnCl_2_ loss, resulting in higher overall reaction energy at −4.29 eV. This pathway is not considered due to no Zn deposition on the surface. Pathway 3 has byproducts of butane and Cl_2_. After Cl_2_ loss, the computed overall reaction energy is −2.00 eV. Pathway 3 has a high energy cost of 4.81 eV to lose Cl_2_. The remaining Cl groups for pathways 1 and 3 can be removed through interaction with another Zn(Et)_2_ reducing agent in the DEZ pulse.

**Figure 8:**
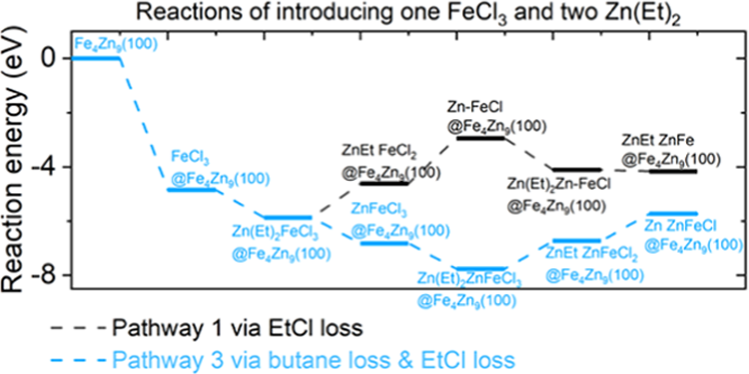
Plotted reaction energy pathways for eliminating the Cl group and the Et ligand via pathways 1 and 3 after introducing two Zn(Et)_2_ reducing agents.

**Figure 9:**
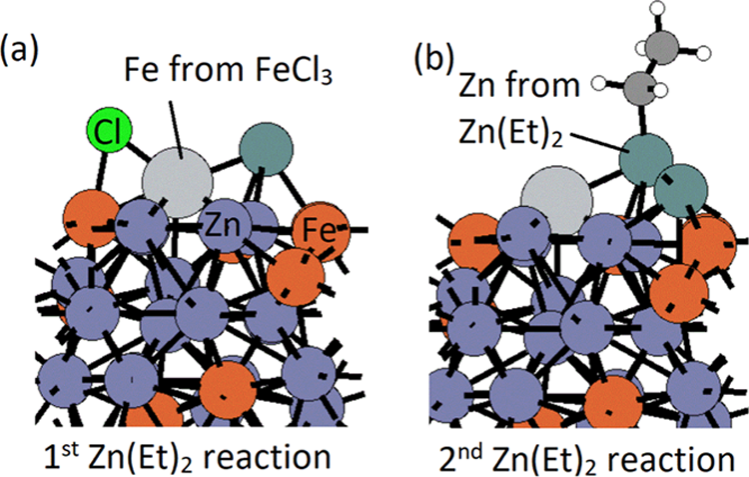
Structures of after ligand elimination of introducing the (a) first Zn(Et)_2_ reducing agent and (b) second Zn(Et)_2_ reducing agent via pathway 1. Substrate Zn and Fe atoms are represented by dark gray and orange colors, Zn and Fe from precursors by dark green and light gray colors, and C, H, and Cl by black, white, and green colors.

After the first Zn(Et)_2_ adsorption and reaction, the surface has one Cl group left for pathway 1 and three Cl groups for pathway 3. We then bring in the second Zn(Et)_2_ reducing agent. As shown in Figure 8, for pathway 1, the second Zn(Et)_2_ has an exothermic interaction energy of −4.11 eV and the loss of the Cl group is exothermic at −4.16 eV. The structures after interaction with two Zn(Et)_2_ are shown for pathway 1 in Figure 9.

**Figure 10:**
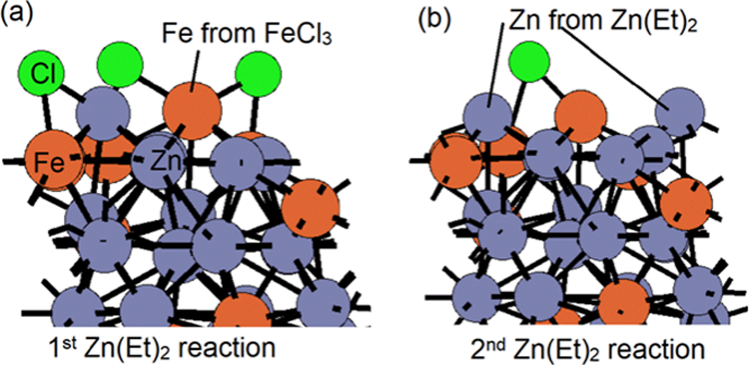
Structures of after ligand elimination of introducing the (a) first Zn(Et)_2_ reducing agent and (b) second Zn(Et)_2_ reducing agent via pathway 3. Zn and Fe atoms are represented by dark gray and orange colors. Cl is represented by green color.

For pathway 3, the second Zn(Et)_2_ has exothermic adsorption energy at −7.76 eV and the loss of two Cl groups via EtCl loss results in exothermic reaction energy at −5.73 eV. The structures after the first Zn(Et)_2_ reaction and the second Zn(Et)_2_ reaction are shown in Figure 10 for pathway 3. These energies are more exothermic than overall reaction energies from pathway 1.

We conclude that the elimination mechanism of the Cl group and the Et ligand is via butane loss and EtCl loss, by introducing one FeCl_3_ and two Zn(Et)_2_. The remaining Cl group or Et ligand will be eliminated through an additional Zn(Et)_2_ in this pulse. The composition of deposited intermetallic Fe–Zn can then be manipulated by controlling the precursor concentration.

## 5 Conclusions

This paper reports a new ALD scheme for the growth of metal alloy/intermetallic thin films. No additional/actual reductant such as hydrogen or hydrides is employed; instead, the two metal precursors react directly to yield both the metal species in a metallic form. Our detailed experimental data are for the intermetallic γ-Fe_4_Zn_9_ phase obtained from the FeCl_3_ + Zn(CH_2_CH_3_)_2_ process. However, our preliminary tests suggested that similar processes could be possibly developed for other metal precursors as well, in combination with DEZ.

From the detailed process parameter investigation for the FeCl_3_ + DEZ process, we could conclude that the process fulfills the basic criteria of an ALD process, that is, the surface-limited and linear (against the number of precursor supply cycles) film growth. Computational DFT calculations indicated that the favorable pathway of eliminating the Cl group and the Et ligand is via butane and subsequent EtCl formation and desorption.

All of the films were crystalline of the γ-Fe_4_Zn_9_ phase, with traces of the α-Fe phase in some samples. Depending on the process parameters, some variation was seen in the degree of crystallinity and also in the Fe/Zn ratio. While not challenged in this work, these intermetallic films could be beneficial, e.g., as biodegradable implant coatings, or in other frontier applications motivated by the simplicity of the fabrication process based on only two well-known ALD precursors. We foresee that the new ALD scheme presented here will trigger the research on the emerging field of high-quality (multi)metal thin films.

## References

[ref1] Suntola T. Atomic layer epitaxy. Mater. Sci. Rep. 1989, 4, 261–312. 10.1016/S0920-2307(89)80006-4.

[ref2] George S. M. Atomic layer deposition: an overview. Chem. Rev. 2010, 110, 111–131. 10.1021/cr900056b. 19947596

[ref3] Leskelä M.; Ritala M. Atomic layer deposition (ALD): from precursors to thin film structures. Thin Solid Films 2002, 409, 138–146. 10.1016/S0040-6090(02)00117-7.

[ref4] Johnson R. W.; Hultqvist A.; Bent S. A brief review of atomic layer deposition: from fundamentals to applications. Mater. Today 2014, 17, 236–246. 10.1016/j.mattod.2014.04.026.

[ref5] Van Bui H.; Grillo F.; van Ommen J. R. Atomic and molecular layer deposition: off the beaten track. Chem. Commun. 2017, 53, 45–71. 10.1039/C6CC05568K. 27725977

[ref6] Zhang Z.; Zhao Y.; Zhao Z.; Huang G.; Mei Y. Atomic layer deposition-derived nanomaterials: oxides, transition metal dichalcogenides, and metal–organic frameworks. Chem. Mater. 2020, 32, 9056–9077. 10.1021/acs.chemmater.9b04414.

[ref7] Richey N. E.; de Paula C.; Bent S. F. Understanding chemical and physical mechanisms in atomic layer deposition. J. Chem. Phys. 2020, 152, 040902 10.1063/1.5133390. 32007080

[ref8] Sønsteby H. H.; Yanguas-Gil A.; Elam J. W. Consistency and reproducibility in atomic layer deposition. J. Vac. Sci. Technol. A 2020, 38, 020804 10.1116/1.5140603.

[ref9] Niemelä J.-P.; Marin G.; Karppinen M. Titanium dioxide thin films by atomic layer deposition: a review. Semicond. Sci. Technol. 2017, 32, 093005 10.1088/1361-6641/aa78ce.

[ref10] Tynell T.; Karppinen M. Atomic layer deposition of ZnO: a review. Semicond. Sci. Technol. 2014, 29, 043001 10.1088/0268-1242/29/4/043001.

[ref11] Tripathi T. S.; Lahtinen J.; Karppinen M. Atomic layer deposition of conducting CuS thin films from elemental sulfur. Adv. Mater. Interfaces 2018, 5, 1701366 10.1002/admi.201701366.

[ref12] Meng X.; Byun Y.-C.; Kim H. S.; Lee J. S.; Lucero A. T.; Cheng L.; Kim J. Atomic layer deposition of silicon nitride thin films: a review of recent progress, challenges, and outlooks. Materials 2016, 9, 1007 10.3390/ma9121007. PMC5457024 28774125

[ref13] Seim H.; Mölsä H.; Nieminen M.; Fjellvåg H.; Niinistö L. Deposition of LaNiO_3_ thin films in an atomic layer epitaxy reactor. J. Mater. Chem. 1997, 7, 449–454. 10.1039/a606316k.

[ref14] Ahvenniemi E.; Matvejeff M.; Karppinen M. Atomic layer deposition of quaternary oxide (La,Sr)CoO_3-δ_ thin films. Dalton Trans. 2015, 44, 8001–8006. 10.1039/C5DT00436E. 25826428

[ref15] Mackus A. J. M.; Schneider J. R.; MacIsaac C.; Baker J. G.; Bent S. F. Synthesis of doped, ternary, and quaternary materials by atomic layer deposition: a review. Chem. Mater. 2019, 31, 1142–1183. 10.1021/acs.chemmater.8b02878.

[ref16] Hämäläinen J.; Ritala M.; Leskelä M. Atomic layer deposition of noble metals and their oxides. Chem. Mater. 2014, 26, 786–801. 10.1021/cm402221y.

[ref17] Hagen D. J.; Pemble M. E.; Karppinen M. Atomic layer deposition of metals: precursors and film growth. Appl. Phys. Rev. 2019, 6, 041309 10.1063/1.5087759.

[ref18] Aaltonen T.; Ritala M.; Arstila K.; Keinonen J.; Leskelä M. Atomic layer deposition of ruthenium thin films from Ru(thd)_3_ and oxygen. Chem. Vap. Deposition 2004, 10, 215–219. 10.1002/cvde.200306288.

[ref19] Martensson P.; Carlsson J.-O. Atomic layer epitaxy of copper growth and selectivity in the Cu(II)-2,2,6,6-tetramethyl-3,5-heptanedionate/H_2_ process. J. Electrochem. Soc. 1998, 145, 2929–2931. 10.1149/1.1838738.

[ref20] Profijt H. B.; Potts S. E.; van de Sanden M. C. M.; Kessels W. M. M. Plasma-assisted atomic layer deposition: basics, opportunities, and challenges. J. Vac. Sci. Technol. A 2011, 29, 050801 10.1116/1.3609974.

[ref21] Tripathi T. S.; Karppinen M. Efficient process for direct ALD of metallic Cu thin films based on an organic reductant. Chem. Mater. 2017, 29, 1230–1235. 10.1021/acs.chemmater.6b04597.

[ref22] Tripathi T. S.; Wilken M.; Hoppe C.; de los Arcos T.; Grundmeier G.; Devi A.; Karppinen M. Atomic layer deposition of copper metal films from Cu(acac)_2_ and hydroquinone reductant. Adv. Eng. Mater. 2021, 23, 2100446 10.1002/adem.202100446.

[ref23] Christensen S. T.; Elam J. W. Atomic layer deposition of Ir-Pt alloy films. Chem. Mater. 2010, 22, 2517–2525. 10.1021/cm9031978.

[ref24] Väyrynen K.; Hatanpää T.; Mattinen M.; Mizohata K.; Meinander K.; Räisänen J.; Link J.; Stern R.; Ritala M.; Leskelä M. Atomic layer deposition of intermetallic Co_3_Sn_2_ and Ni_3_Sn_2_ thin films. Adv. Mater. Interfaces 2019, 6, 1801291 10.1002/admi.201801291.

[ref25] Väyrynen K.; Vihervaara A.; Hatanpää T.; Mattinen M.; Heikkilä M. J.; Mizohata K.; Räisänen J.; Ritala M.; Leskelä M. Nickel germanide thin films by atomic layer deposition. Chem. Mater. 2019, 31, 5314–5319. 10.1021/acs.chemmater.9b01877.

[ref26] Nieminen H.-E.; Kaipio M.; Ritala M. In situ reaction mechanism study on atomic layer deposition of intermetallic Co_3_Sn_2_ thin films. Chem. Mater. 2020, 32, 8120–8128. 10.1021/acs.chemmater.0c01003.

[ref27] Blakeney K. J.; Winter C. H. Atomic layer deposition of aluminum metal films using a thermally stable aluminum hydride reducing agent. Chem. Mater. 2018, 30, 1844–1848. 10.1021/acs.chemmater.8b00445.

[ref28] Lee B. H.; Hwang J. K.; Nam J. W.; Lee S. U.; Kim J. T.; Koo S.-M.; Baunemann A.; Fischer R. A.; Sung M. M. Low-temperature atomic layer deposition of copper metal thin films: self-limiting surface reaction of copper dimethylamino-2-propoxide with diethylzinc. Angew. Chem., Int. Ed. 2009, 48, 4536–4539. 10.1002/anie.200900414. 19444843

[ref29] Vidjayacoumar B.; Emslie D. J. H.; Clendenning S. B.; Blackwell J. M.; Britten J. F.; Rheingold A. Investigation of AlMe_3_, BEt_3_, and ZnEt_2_ as co-reagents for low-temperature copper metal ALD/pulsed-CVD. Chem. Mater. 2010, 22, 4844–4853. 10.1021/cm101442e.

[ref30] Zhong Z.; Wang X.; Ding J.; Yuan N. Nanometer-thick copper films grown by thermal atomic layer deposition. Thin Solid Films 2015, 589, 673–680. 10.1016/j.tsf.2015.06.053.

[ref31] Dey G.; Elliott S. D. Mechanism for the atomic layer deposition of copper using diethylzinc as the reducing agent: a density functional theory study using gas-phase molecules as a model. J. Phys. Chem. A 2012, 116, 8893–8901. 10.1021/jp304460z. 22891810

[ref32] Klug J. A.; Becker N. G.; Riha S. C.; Martinson A. B. F.; Elam J. W.; Pellinaan M. J.; Proslier T. Low temperature atomic layer deposition of highly photoactive hematite using iron(III) chloride and water. J. Mater. Chem. A 2013, 1, 11607–11613. 10.1039/c3ta12514a.

[ref33] Tanskanen A.; Mustonen O.; Karppinen M. Simple ALD process for ε-Fe_2_O_3_ thin films. APL Mater. 2017, 5, 056104 10.1063/1.4983038.

[ref34] Xiang J.; Ding Y.; Du L.; Xu C.; Li T.; Wang X.; Li J.; Zhao C. Investigation of N type metal TiAlC by thermal atomic layer deposition using TiCl_4_ and TEA as precursors. ECS J. Solid State Sci. Technol. 2016, 5, P299–P303. 10.1149/2.0291605jss.

[ref35] Xiang J.; Li T.; Zhang Y.; Wang X.; Gao J.; Cui H.; Yin H.; Li J.; Wang W.; Ding Y.; Xu C.; Zhao C. Investigation of TiAlC by atomic layer deposition as N type work function metal for FinFET. ECS J. Solid State Sci. Technol. 2015, 4, P441–P444. 10.1149/2.0231512jss.

[ref36] Xiang J.; Zhang Y.; Li T.; Wang X.; Gao J.; Yin H.; Li J.; Wang W.; Ding Y.; Xu C.; Zhao C. Investigation of thermal atomic layer deposited TiAlX (X = N or C) film as metal gate. Solid-State Electron. 2016, 122, 64–69. 10.1016/j.sse.2016.04.006.

[ref37] Pore V.; Hatanpää T.; Ritala M.; Leskelä M. Atomic layer deposition of metal tellurides and selenides using alkylsilyl compounds of tellurium and selenium. J. Am. Chem. Soc. 2009, 131, 3478–3480. 10.1021/ja8090388. 19123860

[ref38] Mita K.; Ikeda T.; Maeda M. Phase diagram study of Fe-Zn intermetallics. J. Phase Equilib. 2001, 22, 122–125. 10.1361/105497101770338978.

[ref39] Inui H.; Okamoto N. L.; Yamaguchi S. Crystal structures and mechanical properties of Fe–Zn intermetallic compounds formed in the coating layer of galvannealed steels. ISIJ Int. 2018, 58, 1550–1561. 10.2355/isijinternational.ISIJINT-2018-066.

[ref40] Hashizume Y.; Inomoto M.; Okamoto N. L.; Takebayashi H.; Inui H. Micropillar compression deformation of single crystals of the intermetallic compound Γ-Fe_4_Zn_9_. Acta Mater. 2020, 199, 514–522. 10.1016/j.actamat.2020.08.062.

[ref41] Yadav A. P.; Katayama H.; Noda K.; Masuda H.; Nishikata A.; Tsuru T. Effect of Fe–Zn alloy layer on the corrosion resistance of galvanized steel in chloride containing environments. Corros. Sci. 2007, 49, 3716–3731. 10.1016/j.corsci.2007.03.039.

[ref42] Panagopoulos C. N.; Georgiou E. P.; Agathocleous P. E.; Giannakopoulos K. I. Mechanical behaviour of Zn–Fe alloy coated mild steel. Mater. Des. 2009, 30, 4267–4272. 10.1016/j.matdes.2009.04.026.

[ref43] Kafri A.; Ovadia S.; Yosafovich-Doitch G.; Aghion E. In vivo performances of pure Zn and Zn-Fe alloy as biodegradable implants. J. Mater. Sci. Mater. Med. 2018, 29, 94 10.1007/s10856-018-6096-7. 29938325

[ref44] Kabir H.; Munir K.; Wen C.; Li Y. Recent research and progress of biodegradable zinc alloys and composites for biomedical applications: biomechanical and biocorrosion perspectives. Bioact. Mater. 2021, 6, 836–879. 10.1016/j.bioactmat.2020.09.013. 33024903 PMC7530311

[ref45] Kresse G.; Joubert D. From ultrasoft pseudopotentials to the projector augmented-wave method. Phys. Rev. B 1999, 59, 1758–1775. 10.1103/PhysRevB.59.1758.

[ref46] Perdew J. P.; Chevary J. A.; Vosko S. H.; Jackson K. A.; Pederson M. R.; Singh D. J.; Fiolhais C. Atoms, molecules, solids, and surfaces: Applications of the generalized gradient approximation for exchange and correlation. Phys. Rev. B 1992, 46, 6671–6687. 10.1103/PhysRevB.46.6671. 10002368

[ref47] Perdew J. P.; Burke K.; Ernzerhof M. Generalized gradient approximation made simple. Phys. Rev. Lett. 1996, 77, 3865–3868. 10.1103/PhysRevLett.77.3865. 10062328

[ref48] Monkhorst H. J.; Pack J. D. Special points for Brillouin-zone integrations. Phys. Rev. B 1976, 13, 5188–5192. 10.1103/PhysRevB.13.5188.

[ref49] Libera J. A.; Elam J. W.; Pellin M. J. Conformal ZnO coatings on high surface area silica gel using atomic layer deposition. Thin Solid Films 2008, 516, 6158–6166. 10.1016/j.tsf.2007.11.044.

[ref50] Johansson A.; Ljung H.; Westman S. X-ray and neutron diffraction studies on C-Ni, Zn and C-Fe, Zn. Acta Chem. Scand. 1968, 22, 2743–2753. 10.3891/acta.chem.scand.22-2743.

[ref51] Wilburn D. R.; Bassett W. A. Hydrostatic compression of iron and related compounds: an overview, P = 1. Am. Mineral. 1978, 63, 591–596.

